# Towards a paperless medical physics residency management system

**DOI:** 10.1120/jacmp.v15i6.4866

**Published:** 2014-11-08

**Authors:** Leah K. Schubert, Moyed Miften

**Affiliations:** ^1^ Department of Radiation Oncology University of Colorado School of Medicine Aurora CO USA

**Keywords:** medical physics residency, education, training, documentation

## Abstract

Documentation is a required component of a residency program, but can be difficult to collect and disseminate, resulting in minimal utilization by residents and faculty. The purpose of this work is to adapt a commercially‐available Web‐based medical residency management system to improve the learning experience by efficiently distributing program information, documenting resident activities, and providing frequent monitoring and timely feedback of resident progress. To distribute program information, program requirements and rotation readings were uploaded. An educational conference calendar was created with associated files and attendance records added. To document resident progress, requirements for over 37 different clinical procedures were added, for which the resident logged the total number of procedures performed. Progress reports were created and automatically distributed. To provide feedback to the resident, an extensive electronic evaluation system was created. Results are shown for the initial 21 months of program existence, consisting of a single resident for the first 12 months and two residents for the subsequent 9 months. The system recorded that 130 documents were uploaded and 100% of required documents were downloaded by the resident. In total, 385 educational conferences and meetings were offered, of which the residents attended 95%. The second‐year and first‐year residents logged 1030 and 522 clinical procedures, respectively. The residents submitted a total of 116 status reports detailing weekly activities, 100% of which were reviewed by faculty within an average of 11.3 days. A total of 65 evaluations of the residents were submitted. The residents reviewed 100% of respective evaluations within an average of 1.5 days. We have successfully incorporated a paperless, Web‐based management system in a medical physics residency program. A robust electronic documentation system has been implemented, which has played a central role in enhancing the training experience.

PACS number: 01

## I. INTRODUCTION

Documentation is a required component of a residency program and is necessary for accreditation by the Commission on Accreditation of Medical Physics Educational Programs (CAMPEP, www.campep.org). CAMPEP guidelines list the records required for their review of a program.^(1)^ These records include detailed information about the program itself, including its structure, policies, procedures, objectives, and recruitment files, as well as all activities, achievements, and evaluations each resident has obtained while in the program.

There are obvious challenges to this documentation requirement. An extensive amount of information needs to be documented, which can be time‐consuming to collect, organize, and store. Distributing the evaluations on schedule, ensuring they are collected on time, and providing the resident access to feedback are critical, but can be difficult to achieve in practice. Furthermore, if using a paper record system, there can be limited accessibility.

There are additional challenges specifically for newly created programs. The first challenge is to determine how extensive the documentation system should be. Another challenge is effectively distributing information about the program itself to the rest of the department. There should be good communication to residents and staff about what the program expects the resident to do and how the faculty should be involved, as well as circulation of any updates or changes to program requirements in a timely fashion. Finally, new programs also closely monitor their residents as they continuously improve the learning environment. Therefore, it is even more important for new programs to utilize information about the program and resident progress, which is information that is already required to be collected.

In addition to documentation requirements, physics residency training guidelines provide recommendations on didactic knowledge and skills needed to competently practice medical physics.^2^, ^3^, ^4^ Medical residency training is moving towards a competency‐based approach; however, there is variability in defining, benchmarking, and documenting competency.^5^, ^6^ In the medical physics field, many clinical and quality assurance (QA) procedures need to be performed multiple times in order to gain competency. An initial step in benchmarking competency can be to quantify the minimum number of times a procedure should be performed by a resident. The Accreditation Council for Graduate Medical Education (ACGME), which accredits radiation oncology residency programs, currently has guidelines specifying the required numbers of different cases that medical residents must perform.^(7)^ Current medical physics residency program guidelines leave required numbers to the discretion of the program directors.^1^, ^2^ A data‐driven approach using the information collected by a robust documentation system could provide a starting point in identifying appropriate requirements and eventually identify competencies specific to medical physics.

A small sampling of electronic software programs have previously been compared for use in a physics residency program.^(8)^ This current study details our program's approach to the documentation requirement by turning it into a real‐time learning and communication tool. We describe the comprehensive adaptation of a Web‐based commercially available medical residency management system in order to improve the learning experience by efficiently distributing program information, tracking resident progress, and providing timely feedback.

## II. MATERIALS AND METHODS

A commercially available electronic residency management system (MedHub, Inc., Ann Arbor, MI, www.medhub.com) designed for medical residencies, was adapted for use in a physics residency program. This system is a secure, Web‐based database, accessible for viewing and updating at any time through an Internet connection.

To distribute program information to residents and faculty, the electronic system was used as a central communication, documentation, and data management system. Announcements, reminders, and milestones related to the program were added to the homepage. The electronic system was used to store and provide access to the most current versions of program documents, including the program self‐study document, policies and procedures, and rotation‐specific required readings. Rotation‐specific goals and objectives were also uploaded, which were automatically emailed to the resident and faculty mentor at the start of each rotation.

For resident management, a resident file was created in which demographics, including application materials, new employee forms, and records of achievements such as board certification status, were uploaded. Time‐off requests were initiated by the resident through the electronic system, for which an approval request was automatically emailed. Associated vacation days, conference leave, and sick days were subsequently automatically logged.

To document the resident's activities, multiple features were adapted for use. A progress report form was created using the electronic evaluation feature, to be completed by the resident and viewable by program faculty. This form was automatically sent to the resident on a weekly basis, in which clinical and educational activities were documented. Procedure/case log functionality was used extensively. Throughout the program, the resident is expected to perform various clinical and quality assurance (QA) procedures multiple times. To efficiently document a running total of cases, procedures were added with minimum numbers of times they were required to be performed. Tracking of these procedures was performed on a daily basis and included information about the resident's involvement level (observation, participation, or performance) and involved faculty.

**Figure 1:**
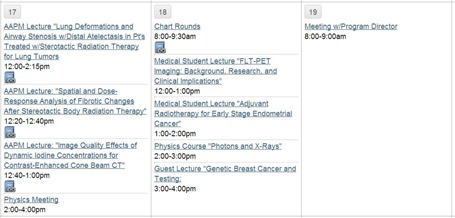
Excerpt of the conference calendar showing required presentations, courses, and department meetings, and physics meetings. Blue icons indicate that files associated with the conferences have been uploaded.

To document the resident's didactic learning activities, the calendar and scheduling features were utilized. Meetings, course lectures, and educational conferences required by the program were added to the calendar, in which resident attendance was recorded. Files associated with presentations, such as journal club articles and presentation slides, were uploaded (excerpt shown in Fig. 1). An automatic tracking feature was used to record the resident's download history.

**Figure 2:**
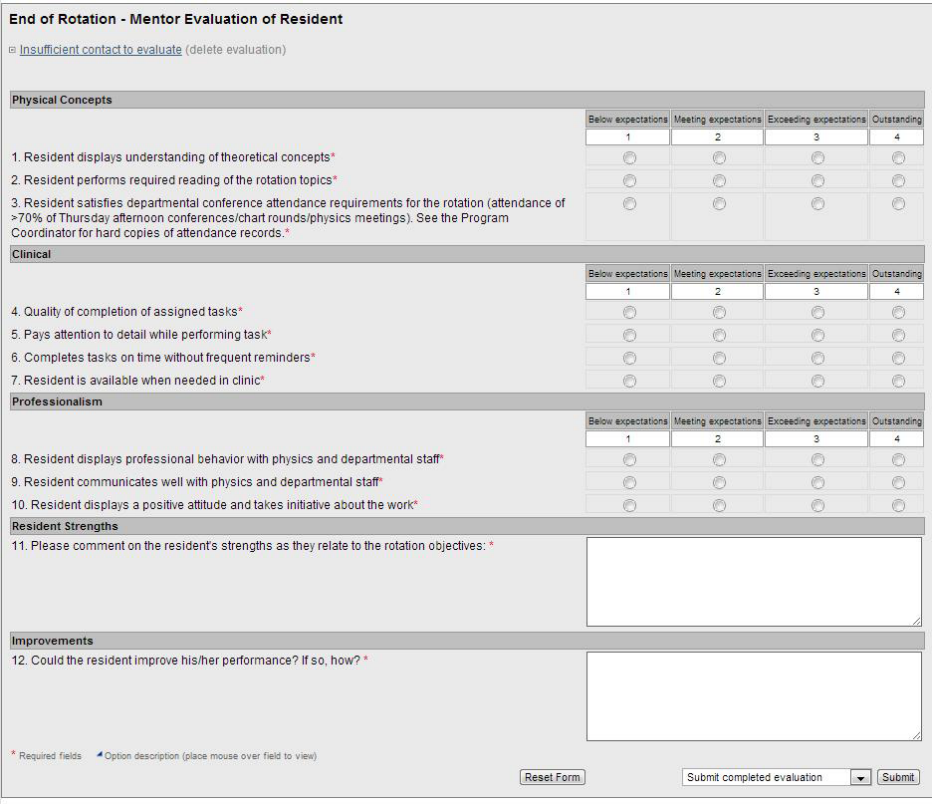
Evaluation form completed by program faculty of the resident at the end of his/her clinical rotation.

The evaluation feature was used for several different purposes. A large number of different electronic forms were created so that each form could be specific while also relatively short and easy to complete. Two electronic report forms were created for residents to report their activities both on‐site and while attending a national conference as part of the program. Two evaluation forms were created for residents to provide written feedback of program faculty and the program itself. Attempts were made to make these forms as anonymous as possible. Resident names were removed from evaluations of program faculty, after which evaluations were aggregated and given to faculty only once per year as part of their annual performance review. For evaluations of the program, the exact same form was given to residents and faculty, which was then made completely anonymous. Finally, to provide feedback to the resident of his/her progress in the program, six different forms were created. Forms were used to evaluate resident's progress at the end of each rotation (shown in Fig. 2) and halfway through each rotation, and following rotation‐specific presentations given to physics faculty, more general presentations given to the department, meetings with faculty on required readings, and oral exams. Email notifications were automatically sent to the recipient when a new evaluation was due, as well as when evaluations were late. In addition to evaluations completed by staff, the resident's progress towards completing select requirements was automatically calculated and displayed for the resident. Progress towards completing procedure requirements were displayed numerically and graphically. Conference attendance records were automatically calculated and displayed against the required attendance percentage.

## III. RESULTS

The medical physics residency program at our institution started in July 2012. Data presented are for the initial 21 months of program existence. The first 12 months consisted of a single resident, while the subsequent 9 months consisted of two residents.

During that time, 18 program announcements were made to the residents and 13 announcements to the faculty on the Web site's homepage. Announcements consisted of topics related to program initiation, accreditation status, structure changes, and recruitment status. A total of 130 documents were uploaded and accessible to residents and faculty. Of those, 15 were related to program policies and expectations, while 115 were rotation‐specific required readings for the resident. An average of 98% of required documents were downloaded by the residents. During the 21‐month period, 385 educational conferences and meetings were offered by the department (average of 4.8 per week). The residents attended an average of 95% of educational conferences and meetings, exceeding the program's required attendance level. The system stored 28 files, including journal club articles and educational presentations, which were uploaded to their respective conferences.

**Figure 3:**
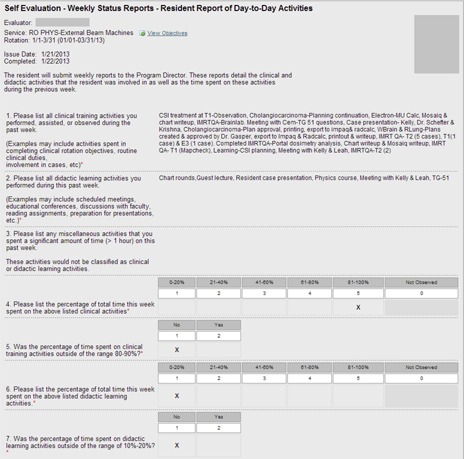
Example of a completed weekly status report. Items in the right column were completed by the resident and describes the clinical and educational activities the resident was involved in during the previous week.

A total of 116 reports documenting the resident's weekly activities were submitted by the residents. An example of a submitted report is displayed in Fig. 3. Faculty reviewed 100% of these reports within an average of 11.3 days (standard deviation 11.7 days).

**Figure 4:**
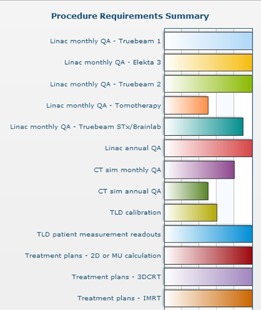
Truncated graphical display of the resident's percentage progress towards completing 13 out of the 37 clinical and QA procedure requirements during the first 21 months in the program. Bars touching the far right side of the graph indicate that the resident has performed the minimum number of procedures to satisfy the requirement.

Requirements for 37 different types of clinical and QA procedures were added. At 21 months in the program, the second‐year resident logged a total of 1030 procedures, of which he observed 12, assisted in 64, and actively performed 954. At eight months in the program, the first‐year resident logged a total of 522 procedures, of which he observed 22, assisted in 25, and actively performed 475. The procedure logs provided clear expectations and a method for the resident to self‐pace and prioritize activities. Additionally they allowed faculty to quickly identify the resident's participation level for specific clinical tasks. Progress toward requirement completion is shown in Fig. 4.

At 21 months in the program, the second‐year resident had completed six rotations and was currently in his seventh rotation. During that time, 47 evaluations providing feedback to the resident were submitted by faculty (average of 2.4 per month). At eight months in the program, the first‐year resident had completed three rotations and was currently in his fourth rotation. During that time, 19 evaluations providing feedback to the resident were submitted by faculty (average of 2.1 per month). One hundred percent (100%) of these evaluations were reviewed by both residents within an average of 1.5 days (standard deviation 2.5 days).

## IV. DISCUSSION

Major goals of using an electronic residency management system are to provide efficient documentation methods and compile information that is well utilized. In our experience, automation tools, easy data entry methods, and instant display of feedback have contributed to the successful achievement of such goals. The large amount of feedback provided to residents via evaluations was made possible by the automatic distribution of electronic forms to the evaluators. Evaluators were automatically sent emails notifying them of new forms to be completed, as well as when evaluations were late. Rather than staff being tasked to distribute and collect paper forms, as well as remind faculty to complete forms on time, the electronic system automatically performed that work. The system was also set up to automatically email rotation goals and objectives at the start of each corresponding rotation to the residents and faculty, thus effortlessly providing notification of program expectations and rotation dates. Automated tools provided efficient monitoring of resident compliance — for example, by calculating the percentage of conferences the residents attended and recording when required readings were downloaded. Easy data entry methods also contributed to the successful utilization of procedure tracking by residents. Rather than requiring residents to keep a free text record, they simply clicked on the procedures performed that day. Since implementation, procedure tracking continues to be well used, which confirms its continued utility. Instant display of feedback is another advantage of electronic systems. Graphing features (example shown in Fig. 4) that automatically calculate progress towards procedure requirements provide instant feedback to the resident. The electronic evaluation forms also provide feedback that is quickly reviewed by residents, which is evident from the average time (1.5 days) it takes the residents to review evaluations from faculty. Feedback has also been used to make changes to the residency program. One of the biggest changes has been the creation of a formal treatment planning lecture series based on resident and faculty annual program review evaluations. Smaller changes include the fine‐tuning of required clinical procedure cases based on the number of cases logged.

There were lessons learned when implementing an electronic documentation system. Redundant or time‐consuming documentation requirements can be burdensome to staff. We initially had two types of evaluation forms documenting monthly meetings, in addition to monthly quizzing sessions over select required readings, both held between the resident and rotation mentor. These forms were somewhat redundant and time‐consuming for faculty to complete every month, so they were shortened and combined into one. Furthermore, the weekly progress reports submitted by the resident have been challenging. The form requires free text summaries of the resident's activities; however, there is duplicate documentation with our use of procedure tracking and conference attendance tracking. We also found that faculty's review of these reports became less timely. During the first eight months of program existence, reports were reviewed in an average of 4.2 days (standard deviation 4.0 days); however, after 21 months in the program, the average increased to 11.3 days (standard deviation 11.7 days). There are several possible reasons for the increased time frame. These particular forms do not have notifications automatically emailed to the faculty when residents have submitted a form, thus reducing their visibility to faculty. Fatigue from reading weekly reports can occur, so a meeting where faculty verbally review activities with the resident is preferred, whenever possible. Finally, using a commercially available system can limit customization options. We currently still use paper checklists to document detailed rotation goals and objectives because we haven't yet found an optimal tool to electronically adapt the checklists to our desired use.

It is important to note that the specific commercial system used in this study, MedHub, has limited availability to those institutions having multiple residency programs and is deployed through the institution's Graduate Medical Education (GME) office. Not all institutions use MedHub, and not all medical physics programs are structured within their institution's GME office. However, if the institution has deployed MedHub, the physics program itself need not be administered through GME, but simply needs permission from their GME office to gain access to MedHub, which was the case for our program. Despite this, potential uses of an electronic documentation system can expand outside of a single residency program and are not dependent on the specific system. Other electronic management systems designed for health‐care education, such as E‐Value (Advanced Informatics, Minneapolis, MN, www.e‐value.net) and Typhon Group systems (Typhon Group LLC, Metairie, LA, www.typhongroup.com) provide tools such as electronic evaluations and/or procedure tracking. A physics residency program need not rely on commercial products to create an electronic documentation system, as one can generate and customize their own internal system using commonly available programming or database tools. Therefore, despite the limited availability of the particular system in this study, the customization, utilization, and experience gained from our implementation of an electronic documentation system can be useful for other physics programs.

Using any electronic system requires upfront effort to establish. For example, electronic evaluations need to be built, electronic forms created and customized, and program documents need to be uploaded. This, however, can be reduced by learning from the experiences of other programs with their own established electronic systems. This sharing of knowledge can potentially improve uniformity in documentation across programs and possibly ease the accreditation process. This would be especially useful for new programs where much effort is spent in forming the structure of the program, while establishing a documentation system may fall to the bottom of the priority list. Currently, there are 67 accredited residency programs in radiation therapy physics, but the majority of those programs (35) have been accredited within the last four years.^(9)^ This indicates a large percentage of newly accredited programs for which this experience in implementing an electronic documentation system could potentially benefit. A centralized electronic documentation system would also apply to programs where residents train at off‐site satellite or affiliated clinics, such as hub and spoke programs. A centralized system is necessary for the central facility to document off‐site resident activities. The ability to access documentation of the resident's progress electronically from any location would also benefit program faculty who rotate between facilities and training duties.

This work has also provided a database of valuable information. Quantifiable data on resident progress is now available, allowing for more objective metrics for benchmarking competency levels. Specifically, the clinical procedure tracking allows the collection of the numbers of different procedures that residents perform. This can be analyzed across different programs, which provides data to potentially establish minimum numbers of procedures required. This system provides the information for a data‐driven approach in establishing guidelines, potentially standardizing and enhancing the quality of physics residency programs.

## V. CONCLUSIONS

A commercially available medical residency management system has been successfully adapted for use in a medical physics residency program. This has played a central role in enhancing the training experience by providing a vehicle for widespread communication of program requirements, documentation of resident activities, and frequent monitoring and feedback of training progress.

A robust electronic system has been implemented in which hundreds of documents have been uploaded and viewed, clinical procedures logged, and educational conferences attended. The use of such a system can provide more robust, uniform documentation across different programs. The data collected by such system can provide information for establishing guidelines which can further standardize and enhance the quality of physics residency programs.

## ACKNOWLEDGMENTS

The authors gratefully acknowledge our institution's Graduate Medical Education office, especially Dr. Carol Rumack, Gail Silber, and Ashley Walter, for providing our program access to the MedHub software. We thank Sandra Korn, our Program Coordinator, who has been instrumental in the success of this system. Finally and most importantly, we thank the current residents, Jerry George, Ph.D. and Joseph John Lucido, Ph.D., for their diligence and extensive use of this documentation system.

## Supporting information

Supplementary Material
