# Supplementary figures and images for: Towards a paperless medical physics residency management system
Source: J Appl Clin Med Phys. 2014 Nov 8;15(6):343–50. doi: 10.1120/jacmp.v15i6.4866 (PMC5711109; doi:10.1120/jacmp.v15i6.4866)

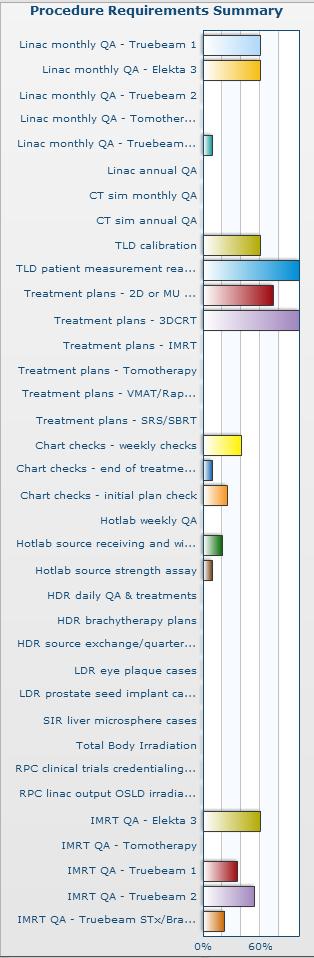

Supplement: Supplementary file 1 — Supplementary Material [file ACM2-15-343-s001.jpg]
